# Efficacy of intravenous lidocaine infusions for pain relief in children undergoing laparoscopic appendectomy: a randomized controlled trial

**DOI:** 10.1186/s12871-020-01218-0

**Published:** 2021-01-05

**Authors:** Maciej Kaszyński, Dorota Lewandowska, Piotr Sawicki, Piotr Wojcieszak, Izabela Pągowska-Klimek

**Affiliations:** 1 Department of Pediatric Anesthesiology and Intensive Care, Medical University of Warsaw University Clinical Centre, ul. Żwirki i Wigury 63A, 02-091 Warsaw, Poland; 2 Department of Anesthesiology and Intensive Care, Division of Teaching, Medical University of Warsaw, ul. Lindleya 4, 02-005 Warsaw, Poland

## Abstract

**Abstract:**

Intravenous lidocaine, a potent local anesthetic with analgesic and anti-inflammatory properties, has been shown to be an effective adjunct that reduces intra- and postoperative opioid consumption and facilitates pain management in adults. While it shows promise for use in the pediatric population, limited evidence is available.

**Objectives:**

To determine if general anesthesia with intraoperative intravenous lidocaine infusion versus general anesthesia without intravenous lidocaine infusion in children undergoing laparoscopic appendectomy decreased opioid requirements intra- and postoperatively.

**Design:**

A single-center parallel single-masked randomized controlled study. A computer-generated blocked randomization list was used to allocate participants. The study was conducted between March 2019 and January 2020. Setting: Pediatric teaching hospital in Poland.

**Participants:**

Seventy-four patients aged between 18 months and 18 years undergoing laparoscopic appendectomy. Seventy-one patients fulfilled the study requirements.

**Intervention:**

Intravenous lidocaine bolus of 1.5 mg/kg over 5 min before induction of anesthesia followed by lidocaine infusion at 1.5 mg/kg/h intraoperatively. The infusion was discontinued before the patients’ transfer to the postanesthesia care unit (PACU).

**Primary outcome measure:**

The primary outcome measure was total nalbuphine requirement in milligrams during the first 24 h after surgery.

**Secondary outcome measures:**

The secondary outcome measures were intraoperative fentanyl consumption, intraoperative sevoflurane consumption, time to the first rescue analgesic request, incidence of postoperative nausea and vomiting during the first 24 h after surgery, frequency of side effects of lidocaine.

**Results:**

Children (*n* = 74) aged 5–17 randomly allocated to receive intraoperative lidocaine infusion (*n* = 37) or no intervention (n = 37). Seventy-one were included in the analysis (35 in the study group and 36 in the control group). There was no difference in the cumulative dose of nalbuphine in the first 24 h after removal of the endotracheal tube between groups [median of 0.1061 (IQR: 0.0962–0.2222) mg/kg in the lidocaine group, compared to the control group median of 0.1325 (IQR: 0.0899–0.22020) mg/kg, *p* = 0.63].

Intraoperative fentanyl consumption was lower in the lidocaine group [median of 5.091 (IQR: 4.848–5.714) μg/kg] than in the control group [median of 5.969 (IQR: 5.000–6.748), *p* = 0.03].

Taking into account the additional doses administered based on clinical indications, the reduction in the requirement for fentanyl in the lidocaine group was even greater [median of 0.0 (IQR: 0.0–0.952) vs 0.99 (IQR: 0.0–1.809) μg/kg, *p* = 0.01].

No difference was observed in the sevoflurane consumption between the two groups [median of 32.5 ml (IQR 25.0–43.0) in the lidocaine group vs median of 35.0 ml (IQR: 23.5–46.0) in the control group, *p* = 0.56].

The time to first analgesic request in the lidocaine group was prolonged [median of 55 (IQR: 40–110) min in the lidocaine group vs median of 40.5 (IQR: 28–65) min in the control group, *p* = 0.05].

There was no difference in the frequency of PONV between the two groups (48.57% in the lidocaine group vs 61.11% in the control group, *p* = 0.29).

No lidocaine related incidence of anaphylaxis, systemic toxicity, circulatory disturbances or neurological impairment was reported, during anesthesia or postoperative period.

**Conclusions:**

Intraoperative systemic lidocaine administration reduced the intraoperative requirement for opioids in children undergoing laparoscopic appendectomy. This effect was time limited, and hence did not affect opioid consumption in the first 24 h following discontinuation of lidocaine infusion.

**Trial registration:**

NCT03886896.

## Introduction

According to current guidelines, intravenous lidocaine infusion emerges as an important component of a multimodal pain management strategy [1, 2]. Intravenous lidocaine, a potent local anesthetic with analgesic and anti-inflammatory properties, has been shown to be an effective adjunct that reduces intra- and postoperative opioid consumption and facilitates pain management in adults. Lidocaine also seems to improve gastrointestinal recovery, reduce postoperative nausea and vomiting, and shorten length of hospital stay [2–4].

While it shows promise for use in the pediatric population [5, 6], limited evidence is available and meta-analyses are inconclusive [7, 8]. This study was planned to evaluate the efficacy of continuous intravenous infusion of lidocaine in reducing opioid consumption during and after laparoscopic appendectomy in children. Intraoperative sevoflurane consumption, time to the first rescue analgesic request, incidence of postoperative nausea and vomiting during the first 24 h after surgery, frequency of side effects of lidocaine was also assessed.

## Methods

### Study design

The study was conducted in a single teaching hospital – the University Clinical Centre of the Medical University of Warsaw.

In this single-blind, randomized controlled trial, children were randomly assigned to two groups according to the use of intraoperative intravenous lidocaine infusions to compare the requirement for opioids during and after laparoscopic appendectomy. Sevoflurane consumption, time to the first nalbuphine dose, incidence of postoperative nausea and vomiting and side effects were also assessed. The study was conducted between March 2019 and January 2020. Patients were enrolled between 26/03/2019 and 15/01/2020.

In accordance with the current Polish law and Declaration of Helsinki the study was approved by the Ethics Committee of the Medical University of Warsaw (KB/24/2019).

The trial was registered at the US National Institutes of Health (ClinicalTrials.gov): NCT03886896. The date of registration: 15/03/2019.

### Study population

Children presenting for laparoscopic appendectomy to be anesthetized by the physicians involved in the study were assessed for eligibility criteria.

The inclusion criteria are listed below:

- Age between 18 months and 18 years;
- ASA physical status class 1E, 2E, 3E;
- Patients undergoing laparoscopic appendectomy.

The exclusion criteria are listed below:

- Allergy to local anesthetics or contraindications for the use of lidocaine;
- ASA physical status class 4E or higher;
- Severe cardiovascular disease;
- Preoperative bradycardia;
- Preoperative atrioventricular block;
- Renal failure;
- Chronic treatment with analgesics;
- Legal guardians’ refusal.

Researchers spoke to the parents or legal guardians and informed them about the study. They described the potential risks and benefits of the procedure, discussed questions and concerns, and then obtained written informed consent.

### Study interventions

**Fig. 1:**
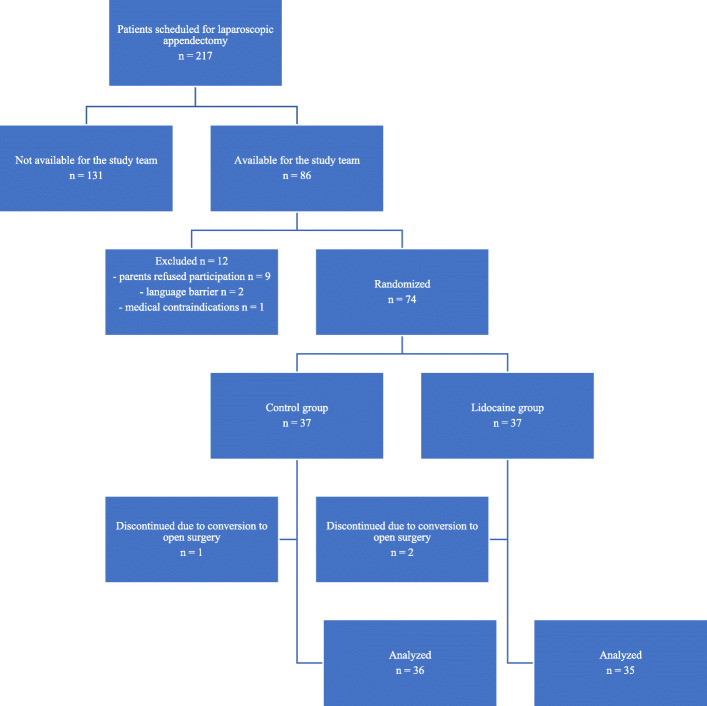
Study flowchart

The enrolment team consisted of three physicians. Only patients operated on during their shifts were evaluated for eligibility criteria (Fig. 1). The other patients were labelled “Not available for the study team”. Participants were randomly assigned to one of two groups.

Patients in the experimental arm received intravenous lidocaine bolus of 1.5 mg/kg over 5 min before induction of anesthesia followed by lidocaine infusion at 1.5 mg/kg/h intraoperatively. The infusion was discontinued before the patients’ transfer to the postanesthesia care unit (PACU).

Patients in the control arm received no additional treatment.

Other than the studied intervention, both groups of participants were treated according to the same fixed perioperative care protocol.

#### Anesthesia protocol description

The peripheral intravenous catheter was inserted in the Emergency Room or in the Surgery Ward when obtaining blood samples. Topical local anesthetics were not used.

Due to the principal diagnosis – acute appendicitis, all of the cases were classified as emergencies (E).

The decision to perform rapid sequence intubation with a high dose of rocuronium was made after individual evaluation based on anamnesis and physical examination carried out by the anesthesiologist. Meeting preoperative fasting recommendations (intervals of 6, 4, and 2 h of fasting for solids, breast milk, and clear fluids, respectively) was one of the factors taken into account in the assessment of the risk of aspiration, but due to the nature of acute abdomen it was of minor importance. Cricoid pressure (Sellick Maneuver) was not utilized.

Upon admission to the operating wing, IV midazolam at 0.05 mg/kg was administered for premedication. The patient was then transferred to the operating theatre, where their vital signs were captured. In the lidocaine group, the loading dose of lidocaine was administered.

Induction of anesthesia was achieved with IV propofol 4 mg/kg, fentanyl 3 μg/kg and rocuronium 0.6–1.2 mg/kg. Intravenous infusion of lidocaine was started in the participants concerned. The second dose of 2 μg/kg fentanyl was administered before skin incision. While under anesthesia, patients were also given acetaminophen 15 mg/kg and metamizole 10 mg/kg. Anesthesia was maintained with sevoflurane. Additional fentanyl doses of 1μg/kg were given when the heart rate or blood pressure exceeded 20% of baseline readings. The minimal alveolar concentration of sevoflurane was titrated to maintain the bispectral index (BIS) in the 40–45 range. A fixed protocol of fresh gas flow (FGF) was utilized. The initial FGF during induction and the first 5 min after intubation was 6 L/min, subsequently reduced to 3 L/min until the vaporizer was closed. The consumption of air and oxygen was used to asses adherence to protocol.

Heart rate, blood pressure, peripheral oxygen saturation (SpO2), BIS, BIS signal quality index (SQI) and electromyography (EMG), total anesthetic gases and anesthetic agent consumption were recorded and archived in the study database. Electrocardiogram, end-tidal carbon dioxide tension, end-expired sevoflurane concentration, gas flow, oxygen concentration, peak and mean airway pressures were monitored as well, however, due to technical limitations, these parameters could not be transferred directly to the database, and some have been archived as photos.

If laparoscopy was converted to open surgery, data collection was discontinued, but pain management strategy was not changed.

After surgery, the children were extubated and transferred to the PACU.

In the PACU, patient monitoring included the heart rate, blood pressure, peripheral oxygen saturation (SpO2), pain, sedation score, nausea and vomiting (Table 1).

**Table 1:** Scales of sedation and PONV used in the study

Sedation scale for PACU		Nausea and vomiting scale	
Score	Description	Score	Description
5	Fully awake	0	Without nausea and Vomiting
4	Drowsy, but awakening with eye contact to voice	1	Nausea
3	No response to voice, but awakening to physical stimulation	2	Vomiting 1 episode per hour
2	Any movement to painful stimuli	3	Vomiting more than 1 episode per hour
1	No response to voice or physical stimulation		

Postoperative pain was assessed using common scales: FLACC (Face – Legs – Activity – Crying – Consolability Scale), NRS (Numerical Rating Scale), or VAS (Visual Analog Scale for pain), depending on the participant’s age and compatibility. When the pain score exceeded 3, nalbuphine titration with an initial dose of 0.1 mg/kg was commenced.

In the surgery ward, non-opioid analgesics were administered on a scheduled around-the-clock (ATC) basis (acetaminophen 15 mg/kg every 6 h and metamizole 10 mg/kg every 8 h). Postoperative pain was assessed every 4–6 h, and on patients’ demand. The nalbuphine dose established previously by titration was administered when the pain score exceeded 3. Incidences of PONV were observed and treated with ondansetron 0.1 mg/kg if necessary.

### Randomization

The eligible children were assigned to groups according to a computer-generated permuted block randomization list prepared by a statistician with no clinical involvement in the trial.

The size of each block was six. Study allocation ratio was 1:1. Information about the participant’s allocation was concealed in a sealed envelope.

During the informed consent process, the researchers, attending anesthesia teams and the children’s families were blinded to treatment allocation. After the consent had been obtained, the investigator opened the envelope. The study drug was prepared by a nurse.

Throughout the study, all medication was administered to randomized patients according to their allocation.

The attending care team at the PACU was not informed about the patients’ treatment allocation, but information about the drugs administered during anesthesia (including lidocaine) was accessible in medical records.

The attending team at the surgery ward was not informed about the patients’ participation in the study prior to one of the researchers coming to the ward to collect the data from medical records (at least 24 h after surgery). No additional information sheet about the patients’ inclusion was attached to medical records. Written information about the trial and the consent forms were given to the participants’ parents or legal guardians, and these were not part of medical documentation. Participants and their families were not informed about the allocation unless they asked.

### Study outcomes

Data were collected in three settings:

1. Operating theatre (OT) by the anesthetist – one of researchers;
2. PACU by one of the researchers if available, or by a regular PACU staff member;
3. Surgical ward (SW) by local nurses.

The primary outcome was postoperative opioid consumption in the first 24 h after removal of the endotracheal tube. Total nalbuphine requirement in milligrams per kilogram body weight was assessed. Data were collected from the patients’ regular medical records.

The secondary outcomes of this study were:

1. The requirement for opioids during anesthesia – assessed by measuring the total amount of fentanyl in micrograms per kilogram body weight used from the induction of anesthesia through to PACU admission.
2. The requirement for volatile anesthetics – assessed by measuring the amount of sevoflurane in milliliters used during anesthesia.
3. Time to first perception of significant pain (time to first rescue analgesic request – defined as pain score > 3 points) – the time to the first dose of nalbuphine that was administered.
4. Incidence of postoperative nausea and vomiting (PONV) in the first 24 h after removal of the endotracheal tube – a four-point ordinal scale was utilized (Table 1).
5. Side effects of lidocaine – assessed by recording the rates of the following complications: arrythmia, hypotension (defined as <70th percentile for age), light-headedness, tinnitus, perioral numbness, during anesthesia and 24 h after removal of the endotracheal tube.

There were no amendments to the study protocol after the commencement of the study.

### Sample size and statistical analysis

Determination of sample size requirement was based on the data available from the study by Lauwick [9]. To detect a minimum 25% reduction in opioid requirement in the postoperative period, with a type-1 error of 0.05 and a power of 80%, the study required 32 subjects in each group.

We allowed for a 15% rate of children lost to follow-up due to conversion to open surgery, need for opioid rotation, incomplete medical records, and other reasons. The final recruitment target of 74 was split into two cohorts of 37 patients each.

Categorical data are expressed as the number of participants and the corresponding percentage of the group. Differences between the proportions of qualitative data were assessed with the χ2 test. Quantitative data were assessed for normal distribution using the Shapiro-Wilk test. Normally distributed data are expressed as mean (SD) and Student’s t-test was used for inter-group comparison. Non-parametric data are reported as median (interquartile range – IQR) and were compared using the Mann-Whitney U test. A *p*-value < 0.05 was considered to be statistically significant. All analyses were conducted using the Statistica software version 13.1 (Statsoft Co.).

## Results

### Study population

Of the 86 patients who were screened, 1 patient did not meet the criteria (ECG abnormalities), 2 patients could not be enrolled due to the language barrier, while 9 patients refused participation. Patient recruitment was suspended from 6 May to 12 May 2019 due to the lack of nalbuphine in the surgery ward.

Seventy-four children met the eligibility criteria and were randomized between March 2019 and January 2020. Seventy-one participants completed the trial. Thirty-five were assigned to the lidocaine infusion group and 36 were assigned to the control group – without lidocaine infusion.

Three patients were excluded due to the conversion from laparoscopic to open surgery.

Baseline demographic and clinical characteristics are presented in Table 2.

**Table 2:** Patients’ characteristics

Variable	Lidocaine group ***n*** = 35	Control group ***n*** = 36	***p***-value
**Gender**			
Female/Male	11/24	11/25	0.94
**Age [years]**			
Median (IQR)	12 (9–13)	12 (9–13.5)	0.87
**Weight [kg]**			
Median (IQR)	40 (28–54)	41 (29–60)	0.73
**ASA Classification**			
IE / IIE	28/7	26/10	0.44

Data are presented in absolute numbers, median (interquartile range – IQR). *P*-values are calculated with χ2 test for qualitative data, and Mann-Whitney U test for quantitative non-parametric data. *ASA* American Society of Anesthesiologists

### Intraoperative clinical data and perioperative non-opioid analgesics consumption

The bispectral index, duration of surgery, duration of anesthesia, time to emerge from anesthesia did not differ between groups (Table 3).

**Table 3:** Intraoperative clinical data

Variable	Lidocaine group ***n*** = 35	Control group ***n*** = 36	***p***-value
**Mean arterial pressure**			
Mean ± SD	68.71 ± 6.51	71.83 ± 7.10	0.07
**Heart rate**			
Mean ± SD	86.31 ± 12.80	90.10 ± 16.03	0.28
**Bispectral index**			
Median (IQR)	44 (42–46)	44 (42–45)	0.23
**Duration of surgery (min)**			
Median (IQR)	58 (48–85)	64 (46.5–89)	0.62
**Duration of anesthesia (min)**			
Median (IQR)	86 (71–114)	90 (74–109.5)	0.39
**Time from the end of surgery (“last strip”) to extubation**			
Median (IQR)	8 (4–12)	8 (5.5–12)	0.79
**Fentanyl before intubation**			
[μg] Median (IQR)	120.0 (90–150)	115.0 (77.5–190)	0.85
[μg/kg] Median (IQR)	3.0 (2.857–3.077)	2.975 (2.857–3.06)	0.81
**Fentanyl before skin incision**			
[μg] Median (IQR)	80.0 (50–100)	80.0 (55–100)	0.94
[μg/kg] Median (IQR)	2.0 (1.852–2.105)	1.961 (1.89–2.067)	0.84
**Cumulative dose of additional fentanyl**			
[μg] Median (IQR)	0.0 (0–30)	40.0 (0–67.5)	< 0.01
[μg/kg] Median (IQR)	0.0 (0.0–0.952)	0.99 (0.0–1.809)	0.01
**Total dose of fentanyl**			
[μg] Median (IQR)	200.0 (160–280)	240.0 (160–337.5)	0.52
[μg/kg] Median (IQR)	5.091 (4.848–5.714)	5.969 (5–6.748)	0.03
**Number of patients who received additional fentanyl dose**			
Number (%)	11 (31.43%)	24 (66.67%)	< 0.01
**Amount of oxygen used**			
[L] Median (IQR)	121.2 (98.4–142.41)	122.98 (101.1–145.9)	0.91
**Amount of air used**			
[L] Median (IQR)	250.96 (214.0–316.22)	255.0 (204.3–327.75)	0.92
**Amount of sevoflurane used**			
[mL] Median (IQR)	32.5 (25.0–43.0)	35.0 (23.5–46.0)	0.56
[mL/h] Median (IQR)	30.53 (25.7–33.3)	30.9 (27.6–36)	0.52

Data are presented in absolute numbers (percentage), mean ± standard deviation – SD, or median (interquartile range – IQR)

Normally distributed data are expressed as mean ± SD and Student’s t-test was used for inter-group comparison. Non-parametric data are reported as median (interquartile range – IQR) and were compared using the Mann-Whitney U test

Doses of Acetaminophen and Metamizole in the whole perioperative period were similar in both groups (Table 4).

**Table 4:** Perioperative non-opioid analgesics and postoperative opioid analgesic requirements

Variable	Lidocaine group ***n*** = 35	Control group ***n*** = 36	***p***-value
**Non-opioid analgesics consumption in the whole perioperative period**			
[mg/kg] Acetaminophen Median (IQR)	75.76 (71.43–77.38)	75.0 (73.25–82.05)	0.63
[mg/kg] Metamizole Median (IQR)	41.46 (40.0–45.98)	40.0 (37.65–44.73)	0.38
**Nalbuphine requirement in all patients**			
[mg] Median (IQR)	5 (2.5–8)	5.5 (2.5–11)	0.64
[mg/kg] Median (IQR)	0.1061 (0.0962–0.2222)	0.1325 (0.0899–0.2202)	0.63
**Nalbuphine consumption in patients who demanded rescue analgesia**			
**Variable**	**Lidocaine group** ***n*** **= 27**	**Control group** ***n*** **= 30**	
**Nalbuphine requirement**			
[mg] Median (IQR)	6 (4–12)	8 (4–12)	0.76
[mg/kg] Median (IQR)	0.1212 (0.1034–0.2791)	0.1951 (0.1–0.3)	0.74
**The time to first analgesic request (to the first dose of nalbuphine)**			
[min] Median (IQR)	55 (40–110)	40.5 (28–65)	0.05

Data are presented as median (interquartile range – IQR)

Non-parametric data are reported as median (interquartile range – IQR) and were compared using the Mann-Whitney U test

### Primary outcome

#### Postoperative opioid consumption in the first 24 h after removal of the endotracheal tube

Of the 71 patients, 51 required nalbuphine in the postoperative period: 27 from the lidocaine group and 30 from the control group. There was no difference in the cumulative dose of nalbuphine in the first 24 h after removal of the endotracheal tube between groups [median of 0.1061 (IQR: 0.0962–0.2222) mg/kg in the lidocaine group, compared to the control group median of 0.1325 (IQR: 0.0899–0.22020) mg/kg, *p* = 0.63].

### Secondary outcomes

#### The requirement for opioids during anesthesia

According to the study protocol, doses of fentanyl administered before intubation and before skin incision were similar in both groups (Table 3).

The number of patients demanding additional doses of fentanyl was different in both groups: 11 (31.43%) in the lidocaine group and 24 (66.67%) in the control group, *p* = 0.003.

Patients in the lidocaine group required less fentanyl intraoperatively (median of 5.091 (IQR: 4.848–5.714) vs 5.969 (IQR: 5.000–6.748) μg/kg, *p* = 0.03).

Taking into account the additional doses administered based on clinical indications (when the heart rate or blood pressure exceeded 20% of baseline readings), the reduction in the requirement of fentanyl in the lidocaine group was even greater [median of 0.0 (IQR: 0.0–0.952) vs 0.99 (IQR: 0.0–1.809) μg/kg, *p* = 0.01].

#### The consumption of volatile anesthetics

No difference in the sevoflurane consumption between the two groups was observed [median of 32.5 ml (IQR 25.0–43.0) in the lidocaine group vs median of 35.0 ml (IQR: 23.5–46.0) in the control group, *p* = 0.56] (Table 3).

#### Time to first perception of significant pain

The time to first analgesic request in the lidocaine group was prolonged [median of 55 (IQR: 40–110) min in the lidocaine group vs median of 40.5 (IQR: 28–65) min in the control group, *p* = 0.05] (Table 4).

#### Incidence of postoperative nausea and vomiting (PONV) in the first 24 h after removal of the endotracheal tube

Thirty-nine of 71 participants suffered from nausea and vomiting: 17 (48.57%) in the lidocaine group and 22 (61.11%) in the control group, *p* = 0.28838.

The differences between groups in the duration of PONV and in the number of patients requiring the use of ondansetron was of no statistical importance (Table 5).

**Table 5:** Postoperative outcome measures

**Variable**	**Lidocaine group** ***n*** **= 35**	**Control group** ***n*** **= 36**	***p*-value**
**PONV**			
number (%)	17 (48.57%)	22 (61.11%)	0.29
**PV**			
number (%)	16 (45.7%)	18 (50.0%)	0.72
**Duration of PONV in patients with PONV**			
Median (IQR)	10.1 (5.13–18.75)	14.2 (8.23–19.33)	0.54
**Patients who received Ondansetron**			
number (%)	8 (22.86%)	12 (33.33%)	0.33
**Dose of ondansetron**			
Median (IQR)	3.5 (3.0–4.0)	4.0 (4.0–4.0)	0.32
**Variable**	**Females** ***n*** **= 22**	**Males = 49**	
**Number of patients who received Ondansetron**			
number (%)	6 (27.27%)	14 (28.57%)	0.91
**Variable**	**Lidocaine group** ***n*** **= 35**	**Control group** ***n*** **= 36**	***p*-value**
**Length of hospital stay after extubation**			
[days] Median (IQR)	4 (4–5)	4.5 (4–6)	0.31
[hours] Median (IQR)	96 (88–116)	109 (87–141.5)	0.41

Data are presented in absolute numbers (percentage), median (interquartile range – IQR)

Non-parametric data are reported as median (interquartile range – IQR) and were compared using the Mann-Whitney U test. PONV – postoperative nausea and vomiting, PV – postoperative vomiting

#### Side effects of lidocaine

No lidocaine related incidence of anaphylaxis, systemic toxicity, circulatory disturbances or neurological impairment was reported, during anesthesia or 24 h after removal of the endotracheal tube.

## Discussion

Lidocaine is an amide local anesthetic primarily used as an antiarrhythmic agent. In 1951 Gilbert et al. published a preliminary report, finding that intravenous lidocaine is safe and effective in relieving severe opiate-resistant pain due to disseminated carcinomatosis and can also produce a useful analgesic effect during labour, forceps delivery, episiotomy, and subsequent repair [10]. Since then, more than 2000 papers have been published demonstrating its benefits: analgesic and antihyperalgesic properties, anti-inflammatory effects, inhibition of bronchial hypersensitivity induced by mechanical irritation, restoring of postoperative bowel function, antithrombotic or even antimicrobial effect [11].

The mechanism underlying the analgesic effect of lidocaine is multifactorial and remains elusive. Lidocaine inhibits sodium, potassium and calcium channels, glycinergic system, NMDA receptors. It can stimulate opioid receptors, decrease excitability and conduction of unmyelinated C fibres, suppress polysynaptic reflexes in the spinal dorsal horn, stimulate muscarinic and nicotinic receptors, and can also act on the cerebral level [10]. In acute neuropathic pain, lidocaine suppresses spontaneous ectopic activity in injured neurons. This property is observed at lidocaine concentration 40 times lower than that required to inhibit nerve conduction [2].

The capacity of lidocaine to alleviate a systemic reaction to surgical trauma may also be due to its anti-inflammatory properties. It inhibits leucocyte adhesion, activation and migration, blocks neutrophil priming, inhibits the release of superoxide anions, attenuates the release of pro-inflammatory cytokines [3, 11].

Similarly, the exact mechanism of the effect of lidocaine on gut motility is not fully understood. According to animal studies, lidocaine may have a direct effect on circular and longitudinal intestinal smooth muscles. Restoring bowel function may be connected with the reduction of pain and opioid consumption [11].

Due to these properties, intravenous lidocaine can play a pivotal role in the concept of Day Surgery and the Enhanced Recovery After Surgery program, especially in abdominal surgery. Despite great interest, the data concerning the benefits of intravenous lidocaine are inconsistent and high-quality studies are still lacking, especially in the pediatric population. The proposed research evaluates the efficacy of perioperative intravenous lidocaine in children undergoing laparoscopic appendectomy.

The study showed that the intraoperative intravenous administration of lidocaine in children undergoing laparoscopic appendectomy was associated with a decrease in intraoperative opiate consumption. The reported effect is consistent with the data observed in other studies [2, 3, 9, 11].

Despite a reduction of the intraoperative opioid requirement, intravenous lidocaine infusion did not significantly reduce postoperative opioid consumption in the study population.

An intravenous lidocaine infusion might affect the postoperative period by increasing the time to the first rescue analgesic request from 40 min in the control group to 55 min in the lidocaine group (*p* = 0.05).

The preventive analgesic effect is commonly considered significant if it extends beyond 5.5 half-lives of the investigated agent, that is for lidocaine approximately 320 min (elimination half-life of lidocaine (t 1/2 beta) 58 min) [12]. Our study did not confirm the mentioned property.

In a study by Alebouyeh et al., lidocaine added to the morphine solution used in patient-controlled intravenous analgesia (PCIA) significantly decreases the pain score and opioid dose after orthopedic surgeries without causing side effects [13].

In light of the findings from the aforementioned paper and other trials investigating prolonged infusions [3, 14], it seems justified to continue systemic lidocaine administration also in the postoperative period, especially in those groups of patients in whom the opioid-sparing effect is particularly indicated. However currently due to the lack of clear evidence, further trials on this issue are required.

Some trials [5, 15] have suggested that intravenous lidocaine accelerates the resolution of postoperative ileus. In our study, however, lidocaine infusion failed to decrease PONV incidence (48.57% in the lidocaine group vs 61.11% in the control group, *p* = 0.29).

The reported frequency of postoperative nausea and vomiting (PONV) in children averages between 33.2 and 82% [16]. The risk factors for PONV can be categorized as those associated with the patient, the type of surgical procedure, and the anesthesia. Eberhart et al. indicated the following clinical risk factors that correlate with postoperative vomiting frequency: duration of surgery ≥30 min, age ≥ 3 years, strabismus surgery, and a positive history of PV in the child, or a history of PONV in parents or siblings [17].

Surgical procedures associated with a high risk of PONV include: strabismus, ear-nose-throat surgery, and appendectomy [16]. In turn, anesthesia-associated risk factors are the use of nitrous oxide and volatile agents, inadequate hydration, high doses of opioids.

In our study, the incidence of PONV was as high as 55%.

Höhne [16] recommended triple PONV prophylaxis for inpatients, older than 3 years of age, undergoing high risk procedures, with surgery time longer than 30 min and predicted frequent use of postoperative opioids.

In the ERAS protocol, aggressive prevention with 2–3 antiemetics in addition to a propofol-based total intravenous anesthetic (TIVA) and opioid-sparing strategies should be encouraged for adult patients at a high risk of PONV [18].

In conclusion, due to the high incidence of PONV in children undergoing laparoscopic appendectomy, antiemetic prophylaxis should be considered.

Intravenous lidocaine infusion, while an effective part of the opioid-sparing strategy, may also be useful in a multimodal antiemetic regimen.

Because of the limited time of action, prolonged infusions should also be considered when used for this indication.

The requirement for hypnotic medication in an adult population was significantly reduced by up to 35% as a result of intravenous lidocaine in a report by Kaba et al. [19] Hamp et al. concluded that a bolus of intravenously administered lidocaine produces a relative reduction of MAC of approximately 12% [20]. We provided an analysis of sevoflurane consumption expressed in milliliters of the utilized agent, and no difference between the studied groups was observed (Table 3).

Volatile anesthetics consumption might be affected by many factors, like the type of agent, duration of administration, agent uptake (patient characteristics, surgical conditions), fresh gas flow, atmospheric pressure, and others [21]. Therefore we managed to prove that lidocaine has no statistically important influence on total sevoflurane consumption which might be of interest from the economic viewpoint. It is worth emphasizing that the results are not comparable with the previously mentioned studies exploring lidocaine influence on patient requirements.

Some authors have suggested that lidocaine infusion might shorten the length of hospital stay [2, 3, 5, 9]. In our study this effect was not confirmed (Table 5), but this outcome measure was not an object of interest and no validated tool supporting hospital discharge decision was utilized.

Due to the risk of inducing systemic toxicity, safety considerations were addressed at the beginning of the study construction. According to the research conclusions of Finholt DA et al., there are no significant differences in the pharmacokinetics of intravenously administered lidocaine during general anesthesia between children older than 6 months of age and adults [12]. El-Deeb et al. studied a protocol with a bolus of 1.5 mg/kg lidocaine administered intravenously followed by infusion at the rate of 1.5 mg/kg/h up to 6 h postoperatively. The studied population of 80 pediatric patients aged 1–6 years had their serum lidocaine levels recorded. Toxic plasma lidocaine level of 5 μg/ml was not reached, and no serious lidocaine-related side effects were reported [5]. Research on healthy volunteers showed that neurotoxic symptoms occur at plasma concentrations above 15 μg/ml while signs of cardiotoxicity occur at plasma concentrations exceeding 21 μg/ml^10^. The aforementioned toxic thresholds are far above the concentrations recorded in El-Deeb’s protocol of infusion administration. In light of these considerations as well as other data available in the literature [6, 22] the proposed scheme of dosage was regarded as safe and chosen for investigation in our study. It was also approved by the Ethics Committee of the Medical University of Warsaw. The scale of potential benefits was described in other sections. Considering the safety profile of intravenous lidocaine infusion at the proposed dose, we decided that the benefits may outweigh the risks.

Our study did not reveal any signs of anaphylaxis, systemic toxicity, circulatory disturbances or neurological impairment. This observation is consistent with other trials [3, 5, 6, 14, 15, 22] investigating the issue.

In the presented study protocol cricoid pressure (Sellick Maneuver) was not implemented. There was no event of tracheal aspiration.

## Conclusions

In conclusion, intraoperative systemic lidocaine administration in the studied pattern reduced the intraoperative requirement for opioids in children undergoing laparoscopic appendectomy. This effect was time limited, and hence did not affect opioid consumption in the first 24 h following discontinuation of lidocaine infusion.

### Study limitations

Single-blinding is the main limitation of the study.

In the study team only three physicians were responsible for providing anesthesia. Only one of them was on duty at the hospital at any one time. He was responsible for participant recruitment, obtaining consent, providing anesthesia, and data collection. Due to the aforementioned circumstances, we concluded that the effort involved in double blinding would further complicate the protocol and might be a source of potential inadequacies in other fields. The measures taken to compensate for the lack of double blinding are described further in this section.

The patients were not informed about allocation. Due to their age and being under general anesthesia, they were obviously unable to consciously affect the measured parameters. The anesthesia team was blinded to group assignment until just before surgery, when the envelope was opened. While selection bias and response bias were prevented, the anesthetist might have, consciously or unconsciously, influenced opioid consumption. To minimize the effects of this bias, the following measures were employed:

- The group of physicians responsible for anesthesia was narrow;
- An explicit protocol of opioid administration was created;
- The researchers strictly adhered to the protocol;

Another limitation is that the effect of intravenous lidocaine infusion on the neuroendocrine response to surgical trauma was not assessed using objective biochemical markers like blood levels of stress hormones and cytokines.

Due to technical limitations, the age-adjusted minimal alveolar concentration (MAC) of sevoflurane, however routinely utilized, was not analyzed. The lack of communication between the anesthetic gases and vapors monitor and the server meant that the data were unavailable for statistical testing. BIS values obtained by the cardiac monitor were continuously sent to the server and are available for further evaluation.

## Data Availability

The datasets used and/or analysed during the current study are available from the corresponding author on reasonable request.
